# Health and wellbeing implications of adaptation to flood risk

**DOI:** 10.1007/s13280-023-01834-3

**Published:** 2023-02-24

**Authors:** Tara Quinn, Stacey Heath, W. Neil Adger, Mumuni Abu, Catherine Butler, Samuel Nii Ardey Codjoe, Csaba Horvath, Pablo Martinez-Juarez, Karyn Morrissey, Conor Murphy, Richard Smith

**Affiliations:** 1 Medical School, University of Exeter, Amory Building, Magdalen Road, Exeter, EX1 2LU UK; 2 Irish Climate Analysis and Research Units (ICARUS), Department of Geography, Maynooth University, Maynooth, Co. Kildare Ireland; 3 School of Psychology, The Open University, Walton Hall, Milton Keynes, MK7 6AA UK; 4 Faculty of Environment, Science and Economy, University of Exeter, Exeter, EX4 4RJ UK; 5 Regional Institute for Population Studies, University of Ghana, Legon Boundary, Accra, Ghana; 6 College of Education, University of Ghana, Legon, P.O. Box LG 1181, Accra, Ghana; 7 Bilbao, Basque Country Spain; 8 Sustainability Division, Department of Technology Management and Economics, Technical University of Denmark, Produktionstorvet, 424, 118, 2800 Kgs. Lyngby, Denmark

**Keywords:** Adaptation, Floods, Health, Typology, Wellbeing

## Abstract

Adaptation strategies to ameliorate the impacts of climate change are increasing in scale and scope around the world, with interventions becoming a part of daily life for many people. Though the implications of climate impacts for health and wellbeing are well documented, to date, adaptations are largely evaluated by financial cost and their effectiveness in reducing risk. Looking across different forms of adaptation to floods, we use existing literature to develop a typology of key domains of impact arising from interventions that are likely to shape health and wellbeing. We suggest that this typology can be used to assess the health consequences of adaptation interventions more generally and argue that such forms of evaluation will better support the development of sustainable adaptation planning.

## Introduction

Globally, the impacts of climate change have become increasingly apparent with current and anticipated events creating imperatives for adaptation measures. In this context, interventions designed to address climate impacts both now and in future have become common in many places around the world. Such developments take multiple different forms and span diverse forms of impact, including floods, ocean acidification, and extreme heat, but they are often assessed and evaluated in similar ways with focus on the extent to which they reduce risk and mitigate economic damage. Within this, rarely is in-depth consideration given to the health and wellbeing implications of the interventions. Rather, many assessments focus on the health and wellbeing benefits derived from risk reduction, but do not consider the breadth of ways in which adaptations might shape and interact with human health (Eakin et al. 2009; Hallegatte et al. 2013; Hammond et al. 2015; O’Brien and Selboe 2015). Though the health and wellbeing implications of implementing climate adaptations are generally under-researched, there are studies that suggest interventions are likely to have important health implications extending well beyond the benefits of impacts avoided. Here, we synthesize evidence from existing literature to give insight into how adaptation interventions themselves can have consequences for health and wellbeing, presenting an analysis that reveals a range of potential areas of direct and indirect impact.

**Fig. 1:**
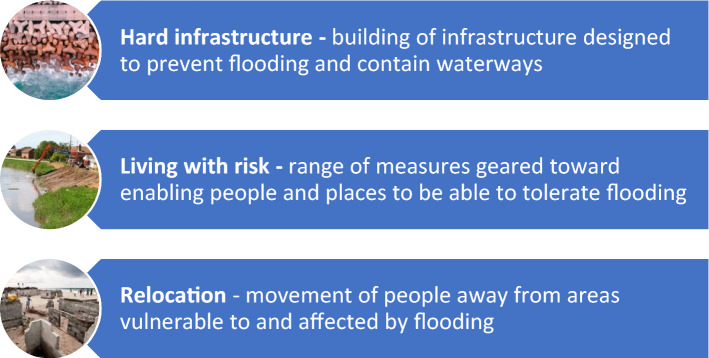
Different strategies for adaptation to flood risk: Hard Infrastructure, Living with Risk, Relocation

This perspective focuses on a major area of climate adaptation underway in multiple places around the world, namely interventions associated with mitigating the increased risk of flooding. The range of experience and research on this form of adaptation makes it possible to synthesize empirical evidence from the literature to develop an understanding of the breadth of domains likely to be affected that have implications for health and wellbeing. There are, of course, multiple possible adaptations to floods that reduce the risk or increase resilience to flooding when it occurs (Adger et al. 2005). For the purposes of the analysis here, we look across three major forms of flood risk adaptation strategy characterised based on practical action taken as well as the underlying rationale or motivation. We term the different forms of adaptive approach; *hard infrastructure*, *living with risk*, and *relocation* (see Fig. 1).

*Hard Infrastructure* (HI) interventions are physical structures such as sea walls and levees that are built with the aim of preventing floodwaters from reaching households and critical infrastructure. *Living with risk* (LWR) reflects a broad group of interventions, at various scales, such as household level adaptations, nature-based approaches, insurance schemes and behavioural change strategies. LWR strategies are undertaken with the aim of adapting to risk and to ensure that houses, communities, and broader regions can withstand, recover, and ultimately thrive in the presence of flood risk; often with individuals undertaking or being affected by multiple adaptation interventions. A growing area of research and practice, for example, focuses on nature-based solutions (NBS) including for example, wetland areas, water-retaining ecosystems and others, with the aim of developing no-regrets solutions for problems such as adaptation, water security and human health (Lin et al. 2021). Such NBSs explicitly seek multiple benefits from “people working with nature, as part of nature, to address societal challenges, providing benefits for both human well-being and biodiversity” (Seddon 2022).

*Relocation* (R) involves moving populations and infrastructure from present locations to new lower-risk locations, either individually or as communities, with the objective of long-term sustainability. The notion of climate-induced relocation is often framed negatively by both policymakers and community members alike and is portrayed as a failure to adapt (IPCC 2018). However, given the increasing understanding of the vulnerability of many communities to future climate-related impacts, relocation is becoming a recognised, viable and sustainable strategy for individual communities and governments when looking to minimise environment-related risk (Black et al. 2011; Marino 2012). Climate-induced relocation is an increasing phenomenon across the globe. Indeed, weather-related displacement is the leading cause of global relocation exceeding that of geophysical displacement 15-fold (IDMC 2013). The option to relocate can be adopted as a planned response to climate-related change, the result of forced displacement due to climate-related emergencies, or part of government-initiated resettlement to enable building and infrastructure.

Each of these strategies of adaptation encompass both anticipatory and reactive action, i.e. actions taken in anticipation of risks or after flood events. The desirability of these interventions can change over time and according to context, with local populations having varying control over the type of interventions that are put forward and realised. While studies such as Dedekorkut-Howes et al. (2020) highlight the predominance of HI flood adaptations globally (see also Harries and Penning-Rowsell 2011; Doberstein et al. 2019), increasingly communities and governments employ the broader suites of adaptation options we outline here. Though each strategy has implications for reducing flood risk, the analysis here suggests that they also have different profiles of impact on people’s lives with implications for the health and wellbeing of local populations.

This perspective draws together empirical evidence across social, environmental, and health sciences relating to these different forms of flood adaptation providing initial insight into the range of potential health and wellbeing consequences. Using the World Health Organisation’s definition of health we consider dimensions of physical, mental, and social wellbeing in our synthesis focusing on the direct impacts of interventions, and also the impacts of the decision-making processes around the implementation of interventions. In concluding, the paper moves to identify a typology to support more comprehensive consideration of health and wellbeing impacts within flood adaptation planning and argues for the inclusion of a wider set of concerns in evaluations of climate adaptation strategies more generally. Ultimately, the paper offers better understanding of the areas of concern for engaging more fully with the health and wellbeing impacts of adaptation processes, suggesting routes to improve outcomes associated with their development and implementation.

## Health and wellbeing consequences of adaptation interventions

The different areas of flood adaptation addressed here are revealing for thinking about the diversity of impacts on the lived experience of affected populations that have implications for health and wellbeing. Beginning with *hard infrastructure*, we use this to refer broadly to large scale engineering solutions for flood risk management, such as physical barriers intended to keep water away from populations and infrastructure. Though they vary in their nature and design, the most well-known examples include raised land around rivers such as bunds, dikes, and levees; walls and embankments built to prevent overtopping and erosion; and dams built to control the flow of water through a catchment. These types of flood defence structure can have positive impacts on health, through reduction of flood risk and associated health risks. Such structures not only protect from direct risk of injury and drowning, but also prevent flooding of key infrastructures ensuring continuity of health services, with proven benefits for rates of infant mortality, for example, in at-risk zones (Myaux et al. 1997). Indeed infrastructure focused adaptations can be an opportunity to improve inadequate infrastructure, helping to address existing inequalities (Sharifi et al. 2021). Research also indicates positive implications for mental health and wellbeing from hard infrastructural works being undertaken (Walker-Springett et al. 2017). However, the literature is revealing for thinking about how hard infrastructural adaptations may also have several more negative potential implications for health and wellbeing.

First, they can alter ecosystems and habitats with subsequent negative implications for the health of affected populations. For example, Kien Nguyen et al. (2019) show that dikes constructed in the Mekong Delta disproportionately affect the nutrition of poorer populations that rely on wild fish as part of their diet. The construction of dams also acts to change ecosystems; changes in flow, river ecology and salinity in proximity to human settlements can result in increased transmission rates of vector borne diseases, including malaria and schistosomiasis (Steinmann et al. 2006), particularly in tropical and subtropical environments (Lerer and Scudder 1999). Finally, a UK study identified the potential risk of sea walls in generating new micro-habitats for mosquitoes where pockets of water (included to counteract the impact of waves on structures) provide the ideal breeding ground for mosquito populations (Medlock and Vaux 2013).

Second, sense of place has been identified as important for wellbeing (Poe et al. 2016) and the changes in landscapes caused by hard flood defences, such as walls along rivers and coastlines, can have implications for processes of attachment and engagement with effects for mental health. Clarke et al.’s (2016) study in Clontarf, Dublin shows how places at risk of flooding are often meaningful for communities in terms of local heritage, recreation, connection to nature, and ultimately therefore, wellbeing. Clarke et al., highlight the importance of incorporating place-based identities into adaptation planning and consultation for wellbeing outcomes and the wider sustainability of interventions. In this case, the negative emotional and mental health impacts of the sea wall on the lived experience of residents undermined the long-term sustainability of the intervention, and ultimately led to a partial, and expensive, roll back. Related to this are studies identifying how disruption of place because of structural defences can also result in solastalgia, a deeply held sadness and grief regarding destruction of valued environments (Higginbotham et al. 2006). For example, Phillips and Murphy (2021) highlight how rock defences in response to coastal erosion in Ireland resulted in loss of a beach and expressions of solastalgia among local residents. These examples of adaptation processes draws attention to the need for adaptation planning to more fully reflect the values of affected populations, in particular highlighting the impact of participations (or non participation) on the effectiveness and fairness of outcomes. Nurhidayah and McIlgorm (2019) show that in Indonesia the legal framework for adaptation to sea level rise does not fully acknowledge the burden of adapting on communities, they suggest that a more inclusive social justice approach is needed increasing involvement of local people in adaptation planning to improve adaptation outcomes. These studies of adaptation interventions in Ireland and Indonesia reflect the broader importance of participation in adaptation planning for wellbeing outcomes (Hügel and Davies 2020).

Several studies have examined the impact of the extensive construction of sea walls in Japan following the 2011 earthquake and Tsunami, after which 440 sea walls were built cumulatively stretching 394 km along Japan’s coastline (Evers 2019; Matanle et al. 2019). A study of public responses in Hokkaido, for example, documents the desire for local communities to resist concrete structures, and the potential to integrate different types of adaptation strategies that reflect concerns about wellbeing and allowing the sightline of the sea to remain. Similarly, populations that had to evacuate following the tsunami had better levels of mental health when they were relocated to areas where forests were part of coastal defences compared to those located near concrete infrastructure (Tashiro et al. 2020). Though disruption of people–place relations and associated impacts on wellbeing have a long history of study (Cunsolo and Ellis 2018), they seem to be underrepresented in the context of climate change and adaptation decision-making (Adger et al. 2011). The initial analysis here is suggestive of the potential importance of disruption of place for health and wellbeing outcomes in the context of adaptations.

There is not yet robust or in-depth empirical evidence on the positive impacts of infrastructure interventions directly on health and wellbeing, beyond the well-established benefits of avoided hazards. There are detailed assessments of the costs of flood impacts themselves, on injury, mortality risks, increased water-borne disease burden in the aftermath of floods, and of mental health burdens of experiencing flooded (Ahern et al. 2005; Wu et al. 2016; Lieber et al. 2022). These significant costs have been documented in every corner of the world and synthesised in meta-analyses and scientific assessments such as the IPCC (Cissé et al. 2022; Harper et al. 2022). Most assessments of adaptation therefore take these impact assessments as the principal or exclusive benefits of flood infrastructure investments. Going forwards, more data on the lived experience of infrastructure-based interventions will help when considering the relative merits of possible adaptation interventions.

The second form of flood adaptation we examine here is what we term *living with risk.* Adaptations that focus on LWR include a range of measures such as increasing absorption of water in upper catchments, household protection at the physical site of flooding, and insurance policies that shape adaptation actions both prior to and after flood events. Nature-based approaches, interventions inspired and supported by nature, are a growing group of LWR strategies and are more broadly used for mitigating greenhouse gas emissions, improving water and air quality and supporting biodiversity. Where nature-based approaches reduce exposure and vulnerability to climate risks, there are numerous benefits for societal health and wellbeing through more secure livelihoods and increased capacity to adapt to change (Seddon et al. 2020). The positive effects of nature and green spaces on health and wellbeing are widely documented (e.g. Cummins and Jackson 2001; Maas et al. 2009; Coutts et al. 2010; De Vries et al. 2013; Richardson et al. 2013; Kopecká et al. 2017; Belčáková et al. 2019; Kumar et al. 2020). Nature-based approaches improve thermal comfort by reducing ambient temperatures during hot days (Lai et al. 2019), reduce air, water and noise pollution (Chiabai et al. 2018; Mancuso et al. 2021) alongside having positive consequences for mental and physical health (Sharifi et al. 2021). There is extensive evidence of the positive impacts of exposure to nature and nature-based approaches for mental health, with green spaces associated with reduced mood disorder treatment (Nutsford et al. 2013) and exposure to higher levels of tree canopy and grass associated with improvements in anxiety and psychological distress levels (Astell-Burt et al. 2013). With a specific focus on flood risk adaptation, work by Hagedoorn et al. (2021) in Vietnam show that nature-based approaches can be the preferred adaptation strategy for vulnerable households that have higher levels of natural resources dependency when they support livelihood options and poverty reduction. All this would suggest the relevance of nature-based approaches to flood adaptation for health and wellbeing, with possibilities for strategies to improve health if they also enhance access to natural environments, introduce blue/green spaces and support more livelihoods (Kolokotsa et al. 2020). More recently, there have been calls to reconsider human-nature relationships in such nature-based approaches, with a more interdependent framing supporting new approaches in planning for healthy landscapes (Welden et al. 2021).

LWR approaches that target the household scale involve a range of actions, including adaptations undertaken to lessen the likelihood of water entering a property (e.g. installing a flood pump); altering a property so that a certain height of floodwater is not disruptive (e.g. giving lower floors over to parking); and making changes to properties that make it easier or quicker to recover (e.g. tiled floors, raised electrical points). Where residents do adopt such measures they tend to install more than one type, and there is evidence that moving valued objects and installing door guards can be associated with reduced negative mental health impacts (Lamond et al. 2015). However, such changes to living spaces can also have implications for an individual’s sense of security, and as outlined in a series of papers by Harries, everyday reminders of flood risk (e.g. raised plug sockets) can be detrimental for mental health, serving as constant reminder of living in an at-risk home (Harries 2008, 2017; Harries and Penning-Rowsell 2011). The evidence thus suggests some complexity in the ways that this type of ‘living with risk’ adaptation approach can affect health and wellbeing, with possibilities for both positive and negative impacts.

Financial mechanisms, such as insurance can support a living-with-risk approach enabling people to stay in place when threatened with flood risk and to recover after a disaster event. When insurance mechanisms are present they can support responses to flood risk, both proactively in providing a sense of security and in the recovery period by providing for the financial cost of repairs. Not having insurance is associated with increased odds of negative mental health outcomes, relative to households with insurance (Mulchandani et al. 2019). However, dealing with insurance companies, and the uncertainty associated with these interactions, has been identified as one of the key drivers of stress and psychological ill health following floods (Carroll et al. 2010; Tempest et al. 2017). Mulchandani et al.’s (2019) study shows that the impacts of interactions with insurance companies is still reflected in mental health outcomes up to two years after a flood event. This is suggestive of the importance of insurance companies’ roles in shaping health and wellbeing in climate adaptation, and identifiable as an area where processes could be designed to lessen negative mental health impacts.

The final form of adaptation increasingly identifiable in response to floods is that of *Relocation*. These strategies aim to draw down risk by moving people, communities, and businesses away from areas vulnerable to flooding (Carey 2020). This includes short-term relocation where people evacuate to move away from immediate danger, the medium-term response of households relocating temporarily while flood hit locations are rebuilt, and the long-term response of planned relocation where communities relocate permanently. Such processes can involve governing bodies and be overseen by authorities or be undertaken by households as part of their responses and they can take many different forms including forcible displacement, resettlement schemes, managed retreat, and migration. The type of relocation has implications for health and wellbeing outcomes of relocating populations.

Research exploring the potential health impacts of these different types of relocation finds that health outcomes are likely to be negatively affected when relocations are forced (McMichael et al. 2012). McMichael et al. find that forced relocations can result in severe and adverse health outcomes for both displaced and host communities including, social instability increasing the risk of sexually transmitted infections such as HIV; adverse health impacts associated with poverty and overcrowding; increases in substance misuse and gender-based violence; and higher rates of depression, social isolation, and loneliness. It is not only forced relocation that have negative wellbeing outcomes, relocation processes that lack transparency can increase distrust and reduce participation, and a financial framing in adaptation decision-making can further entrench historical patterns of social inequity (Siders 2019).

Other research has highlighted how relocation more generally has indirect implications for physical and mental health risks through things such as the loss of land and employment opportunities; loss of shelter; and economic marginalization (De Sherbinin et al. 2011). And changes in the type of ecological and social system for those being relocated have also been shown to be important for health and wellbeing. For example, Panama planners identified how a potential relocation site for the 1000 strong population of the island of Gardi Sugdub could introduce new risks of malaria and yellow fever to the population (Dannenberg et al. 2019).

More broadly, social and cultural impacts such as loss of heritage and ability to maintain cultural rituals or adapt to cultural expectations of host communities, can result in community divisions and potential violence between groups with further knock on effects for health (De Sherbinin et al. 2011; Berkes 2017). In an in-depth longitudinal study of relocated and non-relocated communities, Norris et al. (2004) find that PTSD (post-traumatic stress disorder) and MDD (major depressive disorder) are significantly higher after 6 months in the displaced community, compared to the non-displaced community, with displaced community members specifically stating that they suffered from a lack of community-based identity. This suggests that when communities are displaced, a loss of community identity can reduce abilities to recover from the negative health-impacts of climate-related events.

Whilst there is ample evidence that relocations are related to negative consequences for health, Koslov et al. (2021) draw attention to instances where *supported* relocation can be beneficial. Their study on the impact of buyout schemes on the mental health of people affected by Hurricane Sandy shows that rebuilding can lead to higher levels of stress compared to those who were financially supported to move elsewhere. Importantly, they show how the type of support offered and the process of relocation has implications for the success of these adaptation strategies. Leadership, co-management and participatory processes in relocation projects have also been demonstrated to matter for broader community resilience in new settlements. For example, work by Jamshed et al. (2018) in Pakistan following the extensive floods of 2010 show that NGO led relocation resulted in better community outcomes compared to plans led by government agencies. The authors find that the difference in outcomes in the new settlements was shaped by the NGO focus on livelihoods, skill development, and education opportunities. Similarly, McNamara and Des Combes (2015) found that public involvement in decisions and use of both government and community resources in the relocation of the small island village Vinidogola in Fiji resulted in better school access for children and improved quality of life for residents overall. Ajibade et al.’s (2022) review of managed retreat programmes emphasizes that plans based on equity and justice predict better outcomes for relocated populations than those based on efficiency based metrics. These analyses highlight how it is important to be attentive to the *processes* involved in relocation as an adaptive response to climate change to understand the nature of health and wellbeing impacts.

The evidence discussed here highlight a range of important impacts of flood adaptation interventions for health and wellbeing. Such impacts span different dimensions of social, economic, ecological, and technological change. Through the paper thus far we have sought to offer an indication of the importance of moving beyond a focus on *consequences avoided* within assessments of health and wellbeing, to identify the wide range of implications generated by different forms of adaptation interventions in themselves. In the final section, we move to suggest a way forward for more systematic assessments of the health consequences of adaptation, outlining a typology that reflects and seeks to initially map key areas of impact.

## Taking forward an approach for assessing the health and wellbeing impacts of climate adaptation strategies

To comprehensively evaluate the health and wellbeing impacts of adaptation, we suggest a three-pronged approach that considers the material/economic, social, and environment consequences of interventions (see Table 1). *Material* relates to the tangible, physical aspects of life that a person has access to for meeting everyday needs and how adaptation results in changes to these that, in turn, affect health and wellbeing. These impacts can include ‘public’ and ‘private’ dimensions where ‘private’ refers to assets which are only used/experienced by private individuals (e.g. housing, personal income) and ‘public’ refers to that which is used and experienced, though not necessarily owned, by the general public (e.g. roads and railways).

**Table 1:** Summary of key impacts of adaptation strategies on health and wellbeing structured by material, social, and environment focused metrics

Domains of life impacted by adaptation	Key impacts of adaptation strategies that directly and indirectly shape health outcomes *(proposed metrics)*	Examples from adaptation interventions
Material/Economic *What a person or organisations owns or has access to for meeting their everyday needs. For example, housing, income, food, infrastructure*	Livelihood: *loss/gain of land, effect on employment*	Mård et al. (2019) show how hard infrastructure to prevent flooding increases economic activities in flood risk areas. *Hard Infrastructure*
	Food security: *change in crops, change in diet, malnutrition*	In the Mekong Delta the construction of Dikes has affected access to wild fish, and the diet of local people reliant of fish from common areas (e.g. Kien Nguyen et al. 2019). *Hard Infrastructure*
	Public Infrastructure: *Access to healthcare, protection of key healthcare assets such as hospitals*	Flood embankments in Bangladesh make travel easier, improving the process of referrals and of home visits by community workers (e.g. Myaux et al. 1997). *Hard Infrastructure*
	Insurance coverage: *% people with coverage, cost of insurance, price of excess*	Absence of insurance relates to rates of PTSD (e.g. Mulchandani et al. 2019) Interactions with insurance agencies causing significant stress for flooded householders (e.g., Tempest et al 2017). *Living With Risk*
Social *The elements of personal and relational life that a person has or can have. For example, access to decision-making, security, community connections*	Personal Resilience: *Sense of Security, Anxiety, Sense of Continuity, Happiness, Sense of Coherence*	Installation of household flood resilience measures, such as plug sockets higher up walls, reduces a sense of security as people increasingly identify as at risk and living in an insecure home (e.g. Harries 2008). *Living with Risk*
	Process/Equity: *perception of procedural and distributional justice in decision-making processes; use of commons*	Public involvement in decision-making around relocation of the small Fijian Island of Vinidogola resulted in better school access for children and improved quality of life (e.g. McNamara and Des Combes 2015). *Relocation*
	Community resilience: *relational capital, active belonging, social capital*	Processes of relocation, being led by NGOs or Government organisations, matters for community resilience in new settlements in Pakistan following 2010 floods (e.g. Jamshed et al. 2018). *Relocation*
	Identity: *Social identity, place identity*	The impact of planning restrictions in long-term response to coastal flood risk in on how communities see themselves, and the continuity of their local town. *Living with risk* (Barnett et al. 2021)
Environment *The aspects of a person’s local environment including the physical aspects and the social and cultural meanings that people attach to it. For example, availability of blue/green space, memories associated with a place*	Place: *place meaning, place safety, place attachment, solastalgia*	Place attachment can lead to resistance to adaptation, where adaptations are in contrast to place meanings they can cause mental health problems such as stress and anxiety (e.g. Clarke et al. 2018).* Hard Infrastructure*
	Human/Nature relations: *access to green/blue space, changes to natural environment e.g. wildlife*	Nature-based adaptation approaches are preferred for vulnerable households with high levels of natural resource dependency (e.g. Hagedoorn et al. 2021). *Living with Risk*

We propose a further focus on *social* dimensions including those tied to collective processes or community dynamics and those located more with the individual. This relates to distinctions between personal and community resilience which refer to things experienced at the individual level, such as thoughts and feelings, and those culturally relevant resources in social environments (Ungar and Theron 2019). As identified above, adaptations have impacts on both individual thoughts and feelings and on community and collective life with important implications for health and wellbeing. For example, disruption to sense of community in processes of response to floods has been shown to adversely affect residents’ mental health (Butler et al. 2018). The *process* of adaptation including the presence and nature of community engagement and participation in decision-making is also encompassed within this category. The third dimension of *environment* encompasses the physical and social aspects of place and environment including things like place attachment and cultural meanings reflecting individual and collective processes of place making. This dimension pays attention to the context in which adaptations occur and how adaptations affect people’s relationships with local areas, including human/nature relations. The literature indicates important implications of different adaptations for things like access to nature again with likely effects for health and wellbeing and the success of interventions (Clarke et al. 2016).

These three dimensions, of course, overlap and interact with each other in multiple ways. For example, material and economic dimensions are well known to shape individual and collective resilience (Darlaston-Jones 2007; Quinn et al. 2020). In Table 1, we distil this typology further outlining key metrics and introducing some examples of health and wellbeing impacts that resonate across each dimension. We argue that using this approach offers a means for identifying different forms of health and wellbeing impact along with the relevant underlying processes implicated in shaping them. In separating out economic, social, and environment dimensions, we offer a familiar and resonant way into more systematic consideration of how health and wellbeing is shaped by adaptation processes.

From the analysis here, it is evident that key impacts of adaptations both directly and indirectly shape health and wellbeing outcomes, either through the socio-political processes of adaptation planning or as a result of the consequences of an intervention on wider physical and social determinants of health. The consequences of interventions are complex, and can be both positive and negative, sometimes having unforeseen implications for health and wellbeing (Cheng and Berry 2013; Atteridge and Remling 2018; Eriksen et al. 2021). Additionally, for at-risk populations, it is also likely that several types of adaptations will be implemented concurrently adding further complexity to the issues. Table 1 builds on the evidence synthesized here to outline one way to cut through the complexity and begin to map key areas of impact from adaptation processes that are likely to affect health and wellbeing.

The significance of process for wellbeing outcomes across adaptation interventions brings attention to the need for careful management of adaptation planning. Singh et al. (2022) highlight the role of transparent, accountable, and representative governance and participatory processes in achieving effect adaptation outcomes. Adaptation planning that reflects the everyday reality of diverse populations, that includes representatives and intermediaries across social groups and that encourages a diversity of approaches for engagement and experimentation can lead to more inclusive governance processes (Ziervogel 2019). Indeed, the increasing interest in transformational adaptation that addresses root causes of vulnerability will require inclusive and fair participatory approaches if they are truly going to lead to sustainable adaptations for all (Solecki and Friedman 2021).

## Conclusion

Climate change has been characterised as the biggest global health challenge of the twenty first century (Costello et al. 2009). The challenge of how to maintain and generate sustainable, healthy communities is a central planning issue and key to this challenge is deciding how to adapt to a changing climate. Within this, increases in the frequency and severity of flooding arising from climate change means it represents one of the key areas for adaptation processes and interventions. To date, adaptation strategies to mitigate flooding have mostly focused on the removal of risk as a means to secure positive impacts for health and wellbeing. However, we argue here that rather than just focusing on consequences avoided, it is necessary to fully identify the range of implications generated by different forms of adaptation interventions in themselves.

We suggest that systematically considering health and wellbeing consequences of specific interventions will enable more rigorous and comprehensive information for adaptation planning. This includes considering how adaptation processes themselves can act to create or redistribute vulnerabilities, and the need to include marginalised groups so that effective adaptation reflects health and wellbeing outcomes across populations (Eriksen et al. 2021). Looking towards broader systemic impacts, future research could consider how to engage with public health and risk management experts in developing climate change adaptation plans that focus on health and wellbeing, building evidence from collaborative participatory climate change adaptation projects (Tonmoy et al. 2020). Given the wide scale implementation and importance of adaptations in facilitating sustainable transitions in the coming decades, the need for a fuller understanding of health and wellbeing impacts is thus a critical research and policy imperative.

This synthesis examines three key types of flood adaptation intervention (HI, LWR, and Relocation) and highlights how these are likely to result in significantly different profiles of health and wellbeing outcomes across affected populations but with commonalities in the areas of life impacted and to which it is important to be attentive. It is also clear that impacts on health and wellbeing not only arise from the direct consequences of the interventions, but also emerge from the wider processes, such as interactions with agencies that can exacerbate or cause uncertainty and distress (e.g. timing of evacuations, interactions with loss adjusters, perceived fairness of consultation processes). All this suggests the importance of examining both outcomes and *the process* by which adaptations are undertaken in assessing and understanding the health and wellbeing implications of different interventions.

We have shown that the key health and wellbeing consequences of flood adaptations are related to a range of material, social, and environment impacts. Disaggregating the health consequences in this manner both aligns with existing public health metrics and offers a way into thinking about wellbeing issues tied to specific adaptation processes, rather than simply aggregated and assumed outcomes. It is clear from the synthesis here that the comprehensive impact of adaptations includes consequences beyond only avoidance of flood impacts. Fully identifying health and wellbeing consequences will require the development of decision-making tools that facilitate engagement with these issues through and within adaptation planning efforts. We assert that only by including health and wellbeing dimensions in adaptation decision-making will a more comprehensive understanding of what adaptations mean for communities in the short and long-term be possible.
